# Lightweight semantic segmentation network with configurable context and small object attention

**DOI:** 10.3389/fncom.2023.1280640

**Published:** 2023-10-23

**Authors:** Chunyu Zhang, Fang Xu, Chengdong Wu, Jinzhao Li

**Affiliations:** ^1^Faculty of Robot Science and Engineering, Northeastern University, Shenyang, China; ^2^Shenyang Siasun Robot & Automation Company Ltd., Shenyang, China; ^3^Changchun Institute of Optics, Fine Mechanics and Physics, University of Chinese Academy of Sciences, Beijing, China

**Keywords:** semantic segmentation, lightweight network, context feature enhancement, small object attention, encoder-decoder

## Abstract

The current semantic segmentation algorithms suffer from encoding feature distortion and small object feature loss. Context information exchange can effectively address the feature distortion problem, but it has the issue of fixed spatial range. Maintaining the input feature resolution can reduce the loss of small object information but would slow down the network’s operation speed. To tackle these problems, we propose a lightweight semantic segmentation network with configurable context and small object attention (CCSONet). CCSONet includes a long-short distance configurable context feature enhancement module (LSCFEM) and a small object attention decoding module (SOADM). The LSCFEM differs from the regular context exchange module by configuring long and short-range relevant features for the current feature, providing a broader and more flexible spatial range. The SOADM enhances the features of small objects by establishing correlations among objects of the same category, avoiding the introduction of redundancy issues caused by high-resolution features. On the Cityscapes and Camvid datasets, our network achieves the accuracy of 76.9 mIoU and 73.1 mIoU, respectively, while maintaining speeds of 87 FPS and 138 FPS. It outperforms other lightweight semantic segmentation algorithms in terms of accuracy.

## 1. Introduction

Semantic segmentation is a fundamental yet crucial task in computer vision, aiming to assign a category label to each pixel. This technology has diverse applications, spanning areas such as autonomous driving (Xiao et al., 2023; Yao et al., 2023), remote sensing (Jin et al., 2023; Li et al., 2023), scene analysis (Chen et al., 2022; Sheng et al., 2022), and more. With the assistance of deep convolutional neural networks (CNNs) 7–9, semantic segmentation has made significant progress. However, applying semantic segmentation algorithms still needs to solve numerous challenges. Addressing these issues has become a primary research direction, especially regarding overcoming feature distortion and small object feature loss during network lightweight implementation.

Feature distortion may result in information distortion between the encoded feature representation and the original data, making it challenging for the model to recover the original data from the features accurately. This can lead to poor performance in reconstruction tasks or other tasks relying on encoded features. Numerous neural networks have been proposed to address feature distortion, including Unet (Ronneberger et al., 2015), ResNet (He et al., 2016), and DenseNet (Huang et al., 2017), which introduce skip, residual, and dense connections to alleviate the feature distortion problem. The most effective approach currently is context feature fusion (Chen et al., 2020; Shang et al., 2020; Fan et al., 2021). However, typical context information exchange can only capture predefined fixed spatial range context information and cannot achieve flexible and targeted context information exchange. This paper proposes a long-short distance configurable context feature enhancement module (LSCFEM), which uses configurable context information to enhance features and assign different relevant regions to each pixel. In order to further improve the network speed, we classify and fuse the relevant areas according to the distance from the current features to achieve targeted feature enhancement. The main difference between our designed contextual enhancement module and the above comparison modules is the introduction of learnable feature-related regions, allowing customized enhancement features to be configured for each pixel.

Semantic segmentation of small targets (Kampffmeyer et al., 2016; Yang et al., 2020; Ma et al., 2021) in images has always been a research hotspot in semantic neural networks. Small objects typically have fewer pixels in the image, making them susceptible to being obscured by the background or similar categories, causing difficulty in feature discrimination and learning. Standard improvement methods include high-resolution input (Xu et al., 2020), multiscale processing (Lin et al., 2018), data augmentation (Ma et al., 2019), and postprocessing techniques (Cheng and Liu, 2020). However, these techniques introduce a significant computational burden, making them impractical for lightweight networks. This paper proposes a small object attention decoding module (SOADM) to achieve high-resolution and large-object-guided small object feature recovery by learning the correlation between objects of the same category since objects share feature similarities. This module uses the features of the low-level semantic stage of the same object to enhance the high-level semantic stage and reduce the loss of small object features.

Combining the above design solutions, we designed a lightweight semantic segmentation network (CCSONet) with configurable context and small object attention. The network includes a ResNet backbone, a Long-short distance Configurable Contextual Feature Enhancement Module (LSCFEM), and a small object attention decoding module (SOADM). In order to achieve a lightweight network, we adopt the ResNet backbone for feature encoding. Our CCSONet achieves an accuracy of 76.9 mIoU and 73.1 mIoU on the Cityscapes and Camvid datasets while speed with 87 FPS and 138 FPS, respectively, making it the best lightweight semantic segmentation algorithm currently available in terms of accuracy.

The contributions of this study are as follows:

1. We propose a lightweight semantic segmentation network with configurable context and small object attention (CCSONet) for efficient and high-performance semantic segmentation. It achieves the accuracy of 76.9 mIoU and 73.1 mIoU on the Cityscapes and Camvid datasets, respectively, with speeds of 87 FPS and 138 FPS. It currently outperforms other lightweight semantic segmentation algorithms in terms of accuracy;
2. The long-short distance configurable context feature enhancement module (LSCFEM) enhances the current position’s feature by learning the relevant regions for each pixel. To improve the module’s performance, we adopt a multistage fusion strategy for long and short-range relevant regions, making the context feature fusion more flexible and adaptable, thereby addressing the feature distortion issue during the encoding process;
3. The small object attention decoding module (SOADM) follows the principle of similarity among same-category object features and uses high-resolution features to guide the restoration of small object features. Experimental results demonstrate that this module effectively reduces the feature loss of small objects and improves the network’s segmentation accuracy.

## 2. Related work

### 2.1. Semantic segmentation

Semantic segmentation has become a widely recognized focal point in the academic and industrial sectors, representing a crucial topic in computer vision. The first semantic segmentation method based on deep convolutional neural networks (CNN) is FCN (Long et al., 2015), which has demonstrated outstanding segmentation performance, paving the way for practical applications. In recent years, the field of semantic segmentation has benefited from the application of advanced techniques such as encoder-decoder architectures (Chen et al., 2014, 2017, 2018), recurrent neural networks (Byeon et al., 2015; Liang et al., 2015), and multiscale learning (Chen et al., 2020; Fan et al., 2021) driving its rapid development and bringing new advancements to the field of computer vision (Chen et al., 2016; Liu et al., 2018; Zhang et al., 2019).

Despite the remarkable progress in semantic segmentation, many methods still need help to meet the demands of lightweight processing. In recent years, some researchers have proposed a series of new approaches (He et al., 2021; Sang et al., 2022), which adopt the design principles of network lightweight, aiming to bridge the gap between performance and efficiency. The emergence of these lightweight methods has provided robust solutions for lightweight semantic segmentation, further driving the advancements in this field. PSPNet utilizes pyramid pooling to capture multiscale information by pooling and upsampling feature maps of different resolutions to obtain global and local semantic information. BiSeNet decomposes the segmentation task into two subtasks: a fast path and a detailed path. The fast path processes low-resolution feature maps, while the detailed path handles high-resolution feature maps. Finally, the results of both paths are merged to obtain the final segmentation result. ENet is a lightweight network designed specifically for real-time semantic segmentation tasks. It significantly reduces computation and parameter quantity through carefully designed bottleneck modules and shuffle layers. FastSCNN adopts a lightweight segmentation head and specially designed contextual modules, enabling high-speed inference while maintaining relatively high segmentation accuracy.

### 2.2. Contextual feature fusion

Aims to enhance the representation and performance of the model by fusing information from different levels, modes, or sources. This fusion can be performed at different stages or levels, from shallow to deep features or input to output layers, to adapt to various tasks and data. Mechanisms like pyramid pooling [PSPNet (Zhao et al., 2017)] or dilated convolutions [DeepLab (Chen et al., 2018)] can capture multiscale contextual information, helping better understand objects in an image. SENet (Hu et al., 2018) introduces SE modules through attention mechanisms to adaptively adjust the weights of each channel, enabling context feature fusion of feature channels. DANet (Fu et al., 2019) incorporates bidirectional attention mechanisms, including spatial and channel attention, to fuse multiscale and cross-channel contextual information, thereby improving semantic segmentation performance. CBAM (Woo et al., 2018) combines spatial and channel attention mechanisms, introducing attention modules in each convolution block to adaptively adjust and fuse feature maps. All these methods propagate context information among pixels within a fixed range. This paper allows multi-region information fusion among pixels throughout the entire image. We achieve contextual feature fusion by flexibly configuring paths between pixels to exchange context information.

### 2.3. Small object segmentation

Due to convolution and pooling processes, information about small and fine objects is lost as the network deepens. Several specific methods have been proposed (Liu et al., 2016; Li et al., 2017; Meng et al., 2017; Guo et al., 2018; Yang et al., 2018) to address information loss in small object segmentation. The first approach involves enlarging the input image to enhance the resolution of small objects or generate high-resolution feature maps (Liu et al., 2016; Meng et al., 2017). However, this method, which relies on data augmentation or increasing feature dimensions, often significantly increases training and testing time. The second approach is to develop new variants, such as residual connections (Ronneberger et al., 2015), spatial pyramids (Chen et al., 2017, 2018), and factorized convolutions (Yu and Koltun, 2015), to leverage multiscale feature layers and improve the recognition of objects of different sizes. Although these spatial pyramid structures and factorized convolutions help alleviate this issue, the information in small objects still needs to be improved, making it challenging to use them effectively. The final approach is to utilize postprocessing techniques to enhance the segmentation of small objects, such as postprocessing with Markov Random Fields and Conditional Random Fields (Chen et al., 2018). However, since postprocessing is an independent component of the segmentation model (Guo et al., 2018), this paper proposes a small object attention decoding module (SOADM), which can significantly improve the segmentation of small objects with minimal additional computational cost while possessing strong theoretical interpretability.

## 3. Method

### 3.1. The framework

The introduction of neural networks has driven the rapid development of semantic segmentation, and many efficient semantic segmentation networks have been widely applied in practical engineering. Despite continuous updates in semantic segmentation algorithms, there are still many challenges, particularly in encoding, where feature distortion and small object information loss are particularly prominent. Researchers have proposed various solutions to address the feature distortion issue, with the most effective approach being context information exchange. Common context information exchange methods can only capture context information within predesigned spatial ranges. We propose a long-short distance configurable context feature enhancement module (LSCFEM) to achieve better context information exchange. The issue of small object information loss mainly arises from the reduction of feature resolution caused by convolution and pooling operations, leading to the loss of small object information. Common solutions involve increasing the resolution of small objects by upscaling the input image or generating high-resolution feature maps. However, this strategy can significantly slow down the network’s inference speed, contradicting the lightweight semantic segmentation design principle. To address the above issues, we propose a small object attention decoding module (SOADM), which enhances the feature information of small objects by learning the correlation between large and small objects.

**Figure 1:**
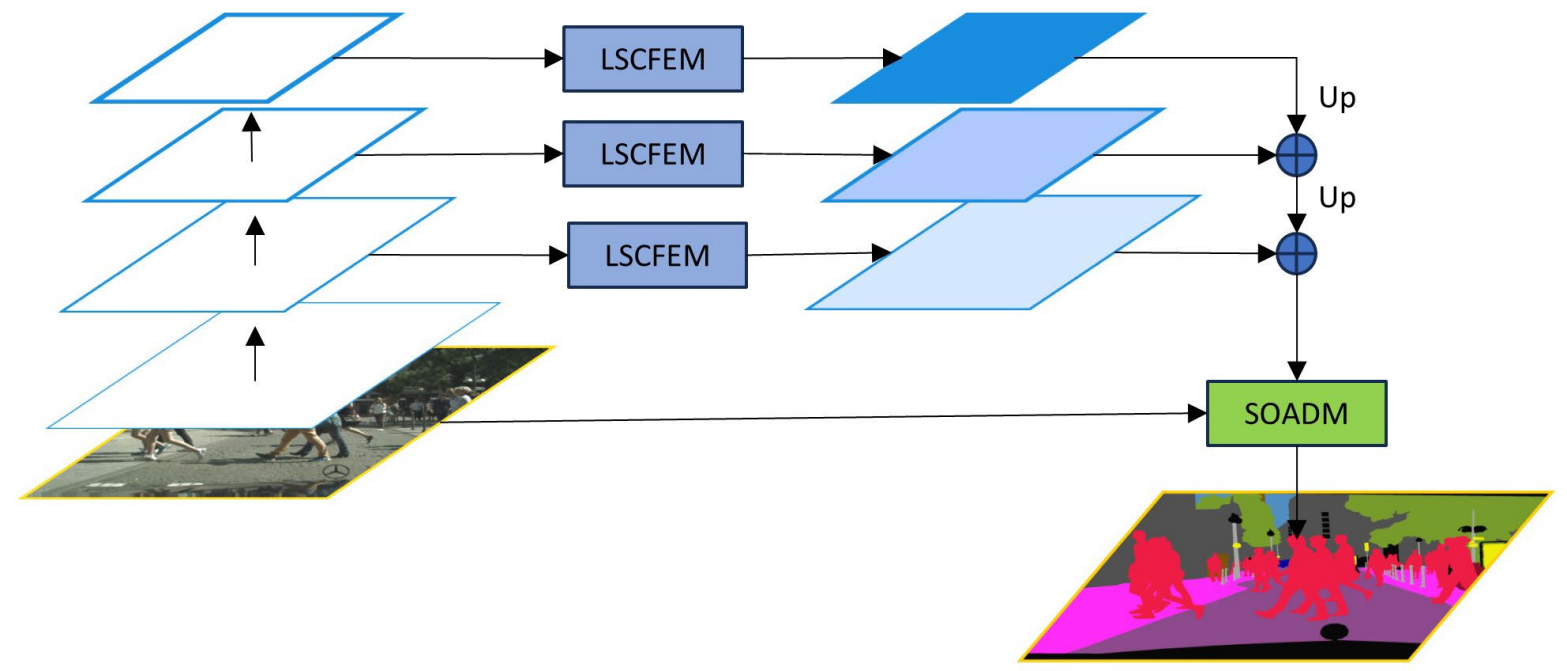
The framework of lightweight semantic segmentation network with configurable context and small object attention (CCSONet) consists of three major components: the Resnet backbone, long-short distance configurable context feature enhancement module (LSCFEM) and small object attention decoding module (SOADM).

Based on the above problem analysis and solution strategies, we present a lightweight semantic segmentation network with configurable context and small object attention (CCSONet). As shown in Figure 1, the framework mainly consists of three modules: the backbone network, the LSCFEM, and the SOADM. We choose ResNet18 as the backbone network to generate hierarchical features in five stages for efficient real-time processing. We select the last three stages’ features and input them into LSCFEM for long-short distance context feature enhancement. The LSCFEM module consists of region segmentation, short-distance correlation feature enhancement, and long-distance correlation feature enhancement, aggregating region information from different stages to enhance target features. Finally, through the small object attention decoding module (SOADM), we model the correlation between classes and increase the capacity of small object features.

### 3.2. The backbone network

In order to strike a balance between performance and efficiency, we chose ResNet18 (He et al., 2016) as the backbone network for extracting features. LEDNet (Wang et al., 2019), a lightweight backbone network, significantly reduces computational costs while maintaining accuracy through innovative operations such as pointwise group convolutions and channel shuffling. Although the computational cost of ResNet18 might be higher than that of LEDNet, the efficient memory access employed by ResNet18 and the lack of fundamental convolutional acceleration in LEDNet make their inference speeds comparable. Moreover, due to ResNet18’s greater parameter count, it possesses superior generalization capability compared to LEDNet. Therefore, ResNet18 is chosen for deployment as the backbone network. ResNet18 generates features across five stages, corresponding to original input resolutions of 1/2, 1/4, 1/8, 1/16, and 1/32. Among these features, only the ones from the third stage (1/8), fourth stage (1/16), and fifth stage (1/32) are utilized as inputs for the LSCFEM. The SOADM requires both the original input image and the final output features of the encoding portion.

### 3.3. The long-short distance configurable context feature enhancement module (LSCFEM)

In the process of coding, the appearance and features of the encoded object can easily result in distortions. Contextual information exchange is currently the best solution. Each pixel needs to be assigned to a specific semantic category in semantic segmentation tasks. Pixels in an image are often influenced by their surrounding pixels, and semantic category correlation exists among them. Therefore, leveraging the semantic information around pixels enables a more precise determination of the semantic category for each pixel. Various neural network components have been proposed to aggregate relevant pixels and construct contextual information. Some representative models include the Spatial Pyramid Pooling (SPP) models (Zhao et al., 2017; Yuan et al., 2018), deformable models (Dai et al., 2017; Deng et al., 2019), and attention models (Hu et al., 2018; Woo et al., 2018; Fu et al., 2019). However, the structures of existing models are predesigned and can only capture category-relevant context within a fixed spatial range.

**Figure 2:**
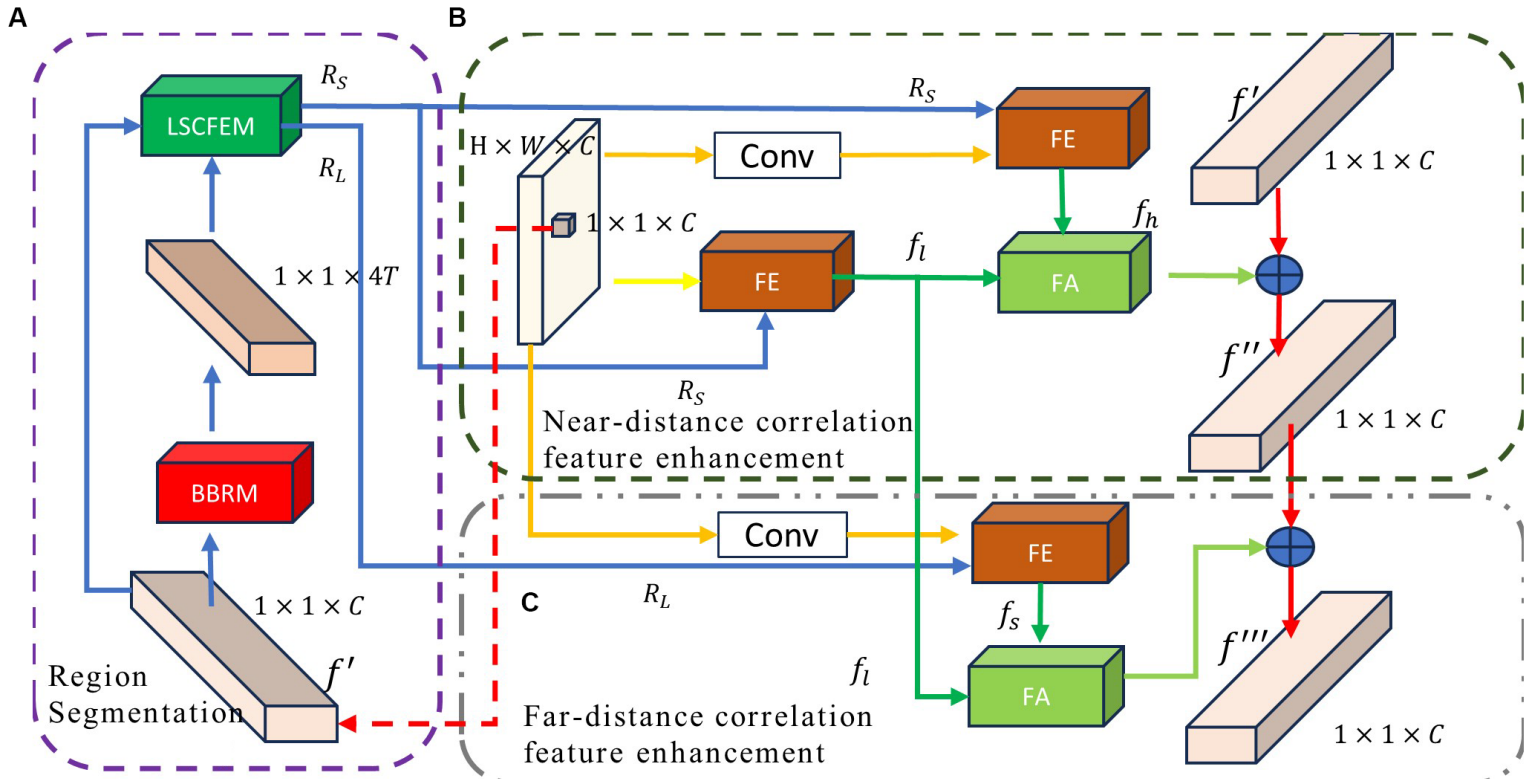
The framework of long-short distance configurable context feature enhancement module (LSCFEM) consists of three major components: **(A)** region segmentation, **(B)** near-distance correlation feature enhancement, and **(C)** far-distance correlation feature enhancement. BBRM: bounding box regression module, FE: feature extraction module, and FA: feature aggregation.

To achieve a more effective context exchange, we propose the long-short distance configurable context feature enhancement module (LSCFEM), a versatile context enhancement model capable of capturing and disseminating context from near and far distances. Previous models did not differentiate between relevant areas based on distance, resulting in feature redundancy during pixel encoding. The LSCFEM constructs near-distance and far-distance relevant features based on the proximity of the target pixel to its associated areas. This module starts by aggregating features from the near-distance region, enhancing the target pixel using the correlated features from the nearby context. The far-distance relevant region features and the near-distance relevant region features exhibit distinct characteristics. For instance, the near-distance relevant region might encompass the object the target pixel belongs to, while the far-distance relevant region could contain objects related to the target object. As such, there is a clear distinction between their features. Therefore, a secondary enhancement and fusion process is performed specifically for the far-distance relevant region features. This module is divided into three main stages: region segmentation, near-distance correlation enhancement, and far-distance correlation enhancement. It enhances target features by aggregating information from different stages, as illustrated in Figure 2. In the phase of region segmentation (as shown in Figure 2A), the feature map is subjected to the boundary box regression module (BBRM) module for relevant boundary extraction. Based on the distance from the current source pixel, the bounding boxes are categorized into near and far-distance bounding boxes. In this stage, relevant near-distance and far-distance regions specific to each pixel are cropped, providing a relevant feature flow to enhance the features of the current pixel. In the near-distance correlation feature enhancement phase (as depicted in Figure 2B), the features of the source pixel are effectively merged and enhanced through the feature extraction from the near-distance relevant regions. Unlike earlier attention models (Liu et al., 2016; Yuan et al., 2018; Xu et al., 2020), where connections between pairs of pixels differ, the feature extraction and merging of the near-distance relevant regions conserve computational resources and simultaneously focus on the crucial context of the nearby relevant regions related to the source pixel. In the far-distance correlation feature enhancement phase (as illustrated in Figure 2C), the far-distance relevant regions are highlighted to expand the relevant context area intentionally. Once again, the features of the far-distance regions are injected into the target pixel, generating the enhanced feature for the target pixel. The specific operations for each stage are as follows.

#### 3.3.1. Region segmentation

**Figure 3:**
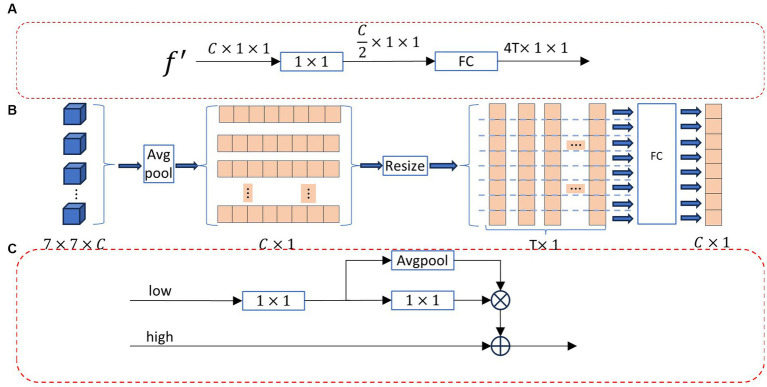
**(A)** bounding box regression module (BBRM), **(B)** feature extraction module (FE), and **(C)** feature aggregation (FA).

In object detection tasks, it is common to localize objects within images. Bounding box regression is a crucial step in object detection, used to predict the position and size of objects. In this paper, we employ bounding box regression to locate the relevant region for the current pixel. Figure 2A shows the region segmentation module, which includes the boundary box regression module (BBRM, Figure 3A) and the long and short region classification function [LSRCF, As shown in Equations (2) and (3)].

BBRM performs bounding box regression through 1×1 convolution and fully connected layers. 1×1 convolution reduces the parameters that need to be learned and increases the depth of the boundary regression model while fully connected layers map feature vectors to predicted bounding box space. Finally, a series of shape adjustment operations are applied to produce a final output tensor of shape [*N, n, 2*], where *N* represents the input batch size, n represents the number of bounding boxes, and the last dimension represents the center of the bounding box, shape is $\left[x_{c},y_{c}\right]$ . The bounding box regression process unfolds as follows:


(1) $$B=Fc\left(\mathrm{Con}v_{1\times 1}\left(F\right)\right)$$

Where Fc represents a fully connected layer, $\mathrm{Con}v_{1\times 1}$ stands for a 1×1 convolution, F denotes the input feature vector, B is the center point of the bounding box with a shape of N×n×2 . It’s worth noting that we normalize all center points and map them onto the current feature map.

Through extensive research, it has been found that the relevant regions for feature enhancement are usually located around the current pixel. However, related area features on the same object will have little enhancement effect on the current features. The purpose of introducing related areas is to ensure that while the pixel enhances the current pixel, other related objects can also contribute to enhancing the current pixel. For example, if the recognized object is a car, the most relevant objects will be roads and other cars, and we can use the characteristics of roads and cars of the same type to enhance the current car. Combining autocorrelation and long-range correlation can result in clearer object boundaries. To this end, we need to classify the relevant areas of the current pixel and fully consider the enhancement of features by long and short-distance related areas. In order to classify the relevant regions of interest into short and long distances, we designed a long and short region classification function (LSRCF). The specific operations of the function are as follows:


(2) $$L_{i}=\sqrt{{\left(x-x_{ci}\right)}^{2}+{\left(y-y_{ci}\right)}^{2}}$$

(3) $$\left(x_{ci},y_{ci}\right)\in \{\begin{array}{c} R_{S}\;\mathrm{if}\;L_{i}\leq M\left(L\right)\\ R_{L}\;\mathrm{if}\;L_{i}>M\left(L\right) \end{array}$$

Where $\left(x_{ci},y_{ci}\right)$ represents the center coordinates of the region of interest, $\left(x,y\right)$ signifies the coordinates of the current encoded pixel, L stands for the collection of distances between the source pixel and all relevant regions, $R_{S}$ and $R_{L}$ denote the near-distance and far-distance relevant regions of the current pixel respectively, and $M\left(\cdot \right)$ represents the median function.

#### 3.3.2. Near-distance correlation feature enhancement

Figure 2B is the structural diagram of the feature extraction module for short-range related areas. This module mainly includes 1 × 1 convolution (Conv), feature extraction module (FE) and feature aggregation module (FA). 1 × 1 convolution is further extracting features from the current feature map to obtain deep features. The FE module combines the feature map and the short-range related area to generate the short-range related area features. FA fuses the close-range correlation region features and the deep close-range correlation region features to generate the final close-range correlation region enhancement features. Finally, the enhanced and original features are summed to obtain the short-range enhanced feature. The operation process of this module is as follows:


(4) $$R_{l}=F_{r}\left(F,\;R_{S}\right)$$

(5) $$F_{S}=\mathrm{Con}v_{1\times 1}\left(F\right)$$

(6) $$R_{h}=F_{r}\left(F_{S},\;R_{S}\right)$$

(7) $$f^{\prime \prime }=A\left(R_{l},\;R_{h}\right)+f^{\prime }$$

Where F represents the input feature map, $\mathrm{Con}v_{1\times 1}$ denotes a 1×1 convolution, $F_{r}\left(\cdot \right)$ signifies the feature extraction operation for the relevant area of interest, $A\left(\cdot \right)$ stands for the module responsible for fusing different hierarchical area features, $f^{\prime }$ is the feature of the current pixel, $f^{\prime \prime }$ represents the neardistance correlation feature, $F_{S}$ stands for deep-level feature, $R_{S}$ denotes the neardistance relevant region of the current pixel, and $R_{l}$ and $R_{h}$ represent the neardistance shallow and deep hierarchical relevant area features, respectively.

The main components of this module are the Feature Extraction (FE) module, as shown in Figure 3B, and the Feature Aggregation (FA) module, as illustrated in Figure 3C. The operational flow of the Feature Extraction module (FE) is outlined below:

Firstly, the near-distance relevant areas are extracted from the input feature map F . The process is as follows:


(8) $$F_{R}=ROI\left(F,\;R_{S}\right)$$

ROI represents the region segmentation operation, $R_{S}$ is the collection of center points for the relevant areas, and $F_{R}$ is the feature map of the relevant areas. Subsequently, $F_{R}$ undergoes global average pooling, computed as follows:


(9) $$F_{X}=Avg\left(F_{R}\right)$$

Where $F_{X}$ is the feature vector of all relevant areas. Finally, a fully connected operation is employed to generate the ultimate region feature vector, with the following calculation:


(10) $$f\;=\;FC\left(\mathrm{Resize}\left(F_{R}\right)\right)$$

Where f is the fused feature of all relevant area features, FC is the fully connected operation, and $\mathrm{Resize}\left(*\right)$ represents feature reorganization, resulting in a shape of $\left[N\times c\times 1\right]$ , where N is the number of input feature maps, and c is the number of channels in the feature map.

The operational flow of the Feature Aggregation module (FA) is as follows:

Firstly, low-level region features are encoded through convolution, computed as follows:


(11) $$F_{1}=\mathrm{Con}v_{1\times 1}\left(F_{l}\right)$$

Where $F_{l}$ stands for the lowlevel region feature, and $F_{l}$ is the intermediate operational feature. Next, the intermediate feature $F_{1}$ undergoes global pooling and a 1×1 convolution operation respectively, as follows:


(12) $$F_{2}=Avg\left(F_{1}\right)$$

(13) $$F_{3}=\mathrm{Con}v_{1\times 1}\left(F_{1}\right)$$

$Avg\left(\cdot \right)$ represents global average pooling. Lastly, $F_{2}$ and $F_{3}$ are elementwise multiplied to generate the final low-level region feature, computed as follows:


(14) $$F_{4}=F_{2}*F_{3}$$

Where ∗ signifies elementwise multiplication. The ultimate output of the Feature Extraction module is obtained through elementwise summation of $F_{4}$ and $F_{h}$ , as follows:


(15) $$f_{a}=F_{4}+F_{h}$$

Where $f_{a}$ is the final output of the Feature Aggregation module, $F_{h}$ represents highlevel region feature, and + denotes elementwise addition.

#### 3.3.3. Far-distance correlation feature enhancement

The operational flow of this module is depicted in Figure 2C. Firstly, convolution is applied to the current feature map to obtain deep-level features at the current position. The deep-level feature map is then subject to feature extraction within the areas of interest, yielding far-distance region features. These far-distance features are then merged with the near-distance features. Finally, the fused far-distance features are summed with the near-distance enhanced features, resulting in the output of the ultimate enhanced feature vector. It is important to note that the output feature is the enhanced feature vector of the current pixel. The operational flow of this module is as follows:

First, feature extraction is performed on the current feature map to obtain deep-level feature $F_{S1}$ :


(16) $$F_{S1}=\mathrm{Con}v_{1\times 1}\left(F\right)$$

Subsequently, based on the center points of the far-distance relevant areas, the deep-level feature is extracted, resulting in far-distance deep-level relevant area feature $F_{h}$ :


(17) $$F_{h}=ROI\left(F_{S1},\;R_{L}\right)$$

$F_{h}$ is then processed through the feature extraction operation $F_{r}\left(\cdot \right)$ for the relevant areas, generating deeplevel relevant area feature $f_{s}$ :


(18) $$f_{s}=F_{r}\left(F_{h}\right)$$

Finally, the far-distance relevant area feature $f_{s}$ and the neardistance relevant area feature $f_{l}$ are input into the feature area fusion module, resulting in the fusion vector $f_{r}$ for near and far distances:


(19) $$f_{r}=A\left(f_{s},\;f_{l}\right)$$

The final enhanced vector output $f^{\prime \prime \prime }$ is obtained by elementwise summation of the near-distance enhanced vector and the fusion vector for far and near distances:


(20) $$f^{\prime \prime \prime }=f^{\prime \prime }+f_{r}$$

Where F represents the input feature map, $\mathrm{Con}v_{1\times 1}$ is a 1×1 convolution, $F_{r}\left(\cdot \right)$ signifies the feature extraction operation for the relevant area of interest, and $A\left(\cdot \right)$ stands for the module responsible for fusing different hierarchical area features.

### 3.4. The small object attention decoding module

The Fully Convolutional Neural Network (Long et al., 2015) (FCN) was first introduced for end-to-end image segmentation, leading to significant advancements in semantic segmentation methods. Although FCN-based semantic segmentation has shown remarkable improvements in segmenting small objects and fine details (Kampffmeyer et al., 2016; Ma et al., 2019; Yang et al., 2020; Ma et al., 2021), challenges persist in segmenting small targets due to the loss of information during convolution and pooling processes. This is because the high-level representations generated through convolutions and pooling reduce the resolution, resulting in the loss of intricate details of small objects. Recovering detailed information about small targets from coarse feature maps is challenging for segmentation models. However, accurately segmenting small objects is crucial in various applications. For instance, in autonomous driving, accurately segmenting and identifying small cars and pedestrians at a distance is of paramount importance. Several methods for segmenting small objects have been proposed. The common strategy is to increase the resolution of small objects by enlarging the input image or generating high-resolution feature maps. Data augmentation or increasing feature dimensions can improve model performance but increase training and testing time. Another promising approach is to develop various network variants, such as skip connections, feature pyramids, and dilated convolutions, to enhance lower-level features. However, this multiscale strategy has certain limitations, as it cannot guarantee feature alignment, and the interpretational power needs improvement, especially for semantic segmentation. Postprocessing techniques like Markov Random Fields and Conditional Random Fields51 can enhance small object segmentation. However, it is important to note that postprocessing is independent of model training, and the network cannot adjust weights based on postprocessing output. Therefore, a comprehensive consideration of these factors is necessary to explore more effective methods for improving the precision of small object segmentation.

We designed the small object attention decoding module (SOADM) to solve the above problems. Its main goal is to use the characteristics of large objects to guide the recovery and recognition of small object characteristics and solve the problem of small object loss. Its operation does not rely on methods such as increasing the dataset size, enlarging the image/feature dimensions, or modifying the network architecture. We observe that objects of the same category often have similar imaging characteristics. We propose exploiting the relationships between small and large objects within the same category to compensate for the feature propagation loss. However, directly calculating the similarity of input images is challenging due to the significant size differences between different objects. Therefore, we propose quantifying this relationship by delving into the feature space. We perform relevant feature extraction on the output of the encoding part and the original image. This step is performed to align the dimensions of small objects in imaging space with the dimensions of large objects in encoded feature space. Subsequently, we utilize small objects in the original image and large objects in the feature map to derive relationships between objects of different sizes within the same category. This is achieved by computing a cross-correlation matrix between intermediate feature patches and image patches, representing the similarity of objects within the same category. Finally, the final decoding features are obtained by adding the relevant features to the upsampled encoding output features.

**Figure 4:**
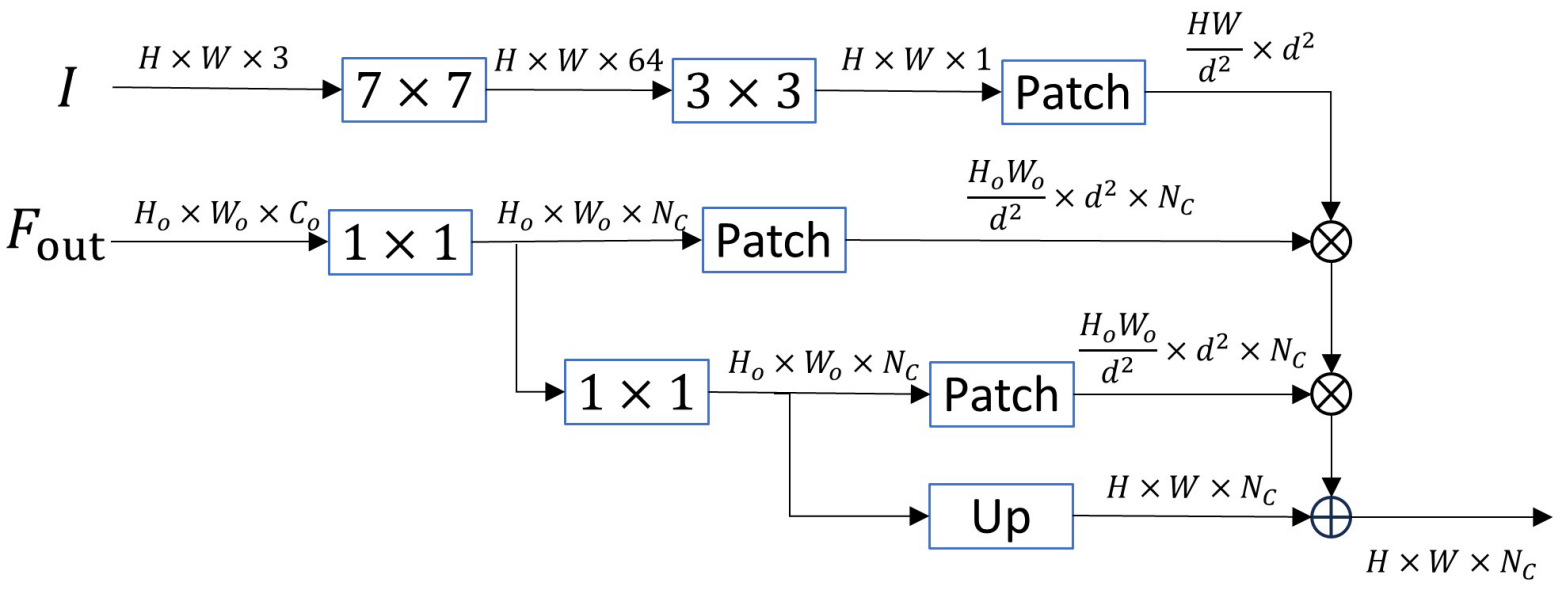
The framework of small object attention decoding module (SOADM). Patch: feature map seg-mentation operation, Up: upsampling operation.

Unlike previous lightweight networks that lack decoders or use simple pyramid decoders, our designed SOADM decoder focuses more on decoding small objects. The pyramid decoder mainly decodes the output of the encoder to deepen the network and improve overall accuracy. However, this approach does not significantly improve the accuracy because much small object information has been lost from the high-level semantic features obtained through multiple downsampling steps. Naturally, decoding features without small object information cannot perform small object segmentation. Our designed small object attention decoding module (SOADM) combines deep semantics with shallow details. It utilizes the relationship features between shallow small and deep large objects to guide the reconstruction of small object features within high-level features. Our decoding module is highly sensitive to small objects and outperforms similar small object attention modules in performance while requiring less computation. It is specifically designed as a decoding module for lightweight semantic segmentation networks. The structure of SOADM is shown in Figure 4. The specific operation process is as follows:

Step 1: Feature extraction is conducted on the original image using 7×7 convolution and 1×1 convolution, resulting in channels of 64 and 1, $F_{1}\in R^{H\times W\times 1}$ respectively. The specific process is as follows:


(21) $$F_{1}=\mathrm{Con}v_{1\times 1}\left(\mathrm{Con}v_{7\times 7}\left(I\right)\right)$$

Step 2: The output feature $F_{\mathrm{out}}$ from the encoding part is processed by two 1×1 convolutions to convert the channel number to the number of segmentation categories and to perform secondary feature decoding. The workflow is as follows:


(22) $$F_{o1}=\mathrm{Con}v_{1\times 1}\left(F_{\mathrm{out}}\right)$$

(23) $$F_{o2}=\mathrm{Con}v_{1\times 1}\left(F_{o1}\right)$$

where $F_{o1}\in R^{H_{i}\times W_{i}\times N_{c}}$ , $F_{o2}\in R^{H_{i}\times W_{i}\times N_{c}}$ , $N_{c}$ represents the predicted number of classes, and each channel contains information about a single category.

Step 3: We segment $F_{1}$ , $F_{o1}$ , and $F_{o2}$ into fixedsize patches. The procedure is as follows:


(24) $$P_{1}=Τ\left(F_{1},\;D\right)$$

(25) $$P_{o1}=Τ\left(F_{o1},\;D\right)$$

(26) $$P_{o2}=Τ\left(F_{o2},\;D\right)$$

where Τ represents image segmentation, segmenting $F_{1}$ , $F_{o1}$ , and $F_{o2}$ into $\frac{HW}{D^{2}}\times D^{2}$ , $\frac{H_{i}W_{i}}{D^{2}}\times D^{2}\times N_{c}$ , and $\frac{H_{i}W_{i}}{D^{2}}\times D^{2}\times N_{c}$ . Each block has a resolution of D×D . Notably, when segmenting $F_{o1}$ and $F_{o2}$ , we perform channelwise feature segmentation and then concatenate the results of all channels.

Step 4: Dot product operations are performed on the blocks of each category in $P_{1}$ and $P_{o1}$ to enhance the correlation between each patch of the original image and each category in $F_{\mathrm{out}}$ . The operation is as follows:


(27) $$A_{1}=P_{1}⊗P_{o1}$$

where $A_{1}\in R^{\frac{HW}{D^{2}}\times \frac{H_{i}W_{i}}{D^{2}}N_{c}}$ represents the correlation between each patch of the original image and each category.

Step 5: The associated features obtained above are correlated with the deep features of $F_{\mathrm{out}}$ to restore the features to the initial resolution of H×W . The operation is as follows:


(28) $$A_{2}=A_{1}⊗P_{o2}$$

(29) $$A=T^{-1}\left(A_{2}\right)$$

where $A_{2}\in R^{\frac{HW}{D^{2}}\times D^{2}\times N_{c}}$ , $A\in R^{H\times W\times N_{c}}$ .

Step 6: Finally, the $F_{o2}$ features are upsampled to match the resolution of H×W . The enhanced feature A is summed elementwise with the upsampled feature $F_{u}$ to produce the network’s final decoding feature out. The procedure is as follows:


(30) $$F_{u}=U\left(F_{o2}\right)$$

(31) $$\mathrm{Out}=A+F_{u}$$

where $F_{u},\mathrm{Out}\in R^{H\times W\times N_{c}}$ . Equation (31) adds upsampled features $F_{u}$ and enhanced features A one by one to reduce the problem of feature offset caused by feature upsampling and improves the recognition accuracy of the network.

### 3.5. The loss function

In order to address the issue of imbalanced class distribution in natural images, we have employed the Online Hard Example Mining technique and a weighted loss method46 to enhance the learning effectiveness of our model further. In our approach, the loss function utilizes cross-entropy error to compute the class for each pixel. During the loss calculation, we sort the pixels based on the cross-entropy loss and then backpropagate errors from the top N positions in the ranking. It is important to note that, for efficient training, we consider only the top 50% of the total pixel count, denoted as E, for loss calculation. Additionally, we introduce a weighted strategy for handling pixel loss, assigning weights based on the pixel proportion of each category. This weighted loss emphasizes handling small objects, thus avoiding the problem of classifier overfitting caused by imbalanced data distribution. We represent the weight vector as $w\in R^{K}$ , where it is calculated on the dataset and K represents the number of classes. Specifically, the definition of the weighted cross-entropy loss is as follows:


(32) $$l=-\frac{1}{N}\overset{E}{\underset{i=1}{\sum }}\omega \left[y_{i}\right]\cdot \prod \left[h_{i}^{y_{i}}<t_{N}\right]\cdot \log h_{i}^{y_{i}}$$

Where $h_{i}^{y_{i}}={\hat{H}}^{y_{i}}\left(i\right)$ represents the difference between the predicted posterior probability of pixel $i\in \left(H\times W\right)$ and its corresponding target class label $y_{i}$ .

## 4. Experiments

### 4.1. Datasets and performance metrics

We detailedly evaluated our CCSONet on two well-known city street datasets. Firstly, the CamVid (Brostow et al., 2009) dataset consists of 367 training images, 100 validation images, and 233 testing images, covering 11 different categories. It is worth noting that, according to the object size definition (Ronneberger et al., 2015), we classify identifiers pedestrians, lampposts, and bicycles as small objects. In contrast, the other seven object categories are classified as large objects. This subdivision helps better consider the characteristics of different object sizes.

Another dataset we used is Cityscapes (Cordts et al., 2016). Cityscapes includes 5,000 annotated images, with 2,975 for training, 500 for validation, and 1,525 for testing. It is important to emphasize that in this paper, we only considered fine-grained annotations for training to ensure high-quality learning of the model. The Cityscapes dataset covers a total of 19 semantic classes. Similarly, based on object size classification, we defined categories such as lampposts, traffic lights, traffic signs, pedestrians, cyclists, motorcycles, and bicycles as small object categories. In contrast, the other 12 object categories are designated as large object categories.

To measure the performance of our method, we adopted the category Intersection over Union (IoU) and mean Intersection over Union (mIoU) as the evaluation metrics for segmentation performance.

### 4.2. Implementation details

Our study employed a training approach from scratch for the segmentation tasks on the CamVid and Cityscapes datasets without utilizing ImageNet pre-trained backbone networks, as indicated in the reference. Throughout the experimentation, we meticulously adhered to the model configuration as outlined in the original text. Our experiments did not involve using any roughly annotated images or additional data.

The model training was conducted using the PyTorch framework, employing a minibatch stochastic gradient descent (SGD) optimization algorithm with a momentum of 0.9 and weight decay of 5e-4, coupled with an adaptive learning rate strategy. The batch sizes were 16 for CamVid and 8 for Cityscapes datasets. To augment the data, we applied techniques such as random horizontal flips (with a probability of 0.6), random cropping, and random scaling (within the range [0.75, 2.0]). For CamVid, the cropped resolution was set to 480×640, while for Cityscapes, the cropped resolution was 640×800 .

During the model training process, we performed 200 epochs of training on the CamVid dataset and 500 epochs on the Cityscapes dataset. The initial learning rate was set to 10e3, which was reduced by 10 after the 200th, 300th, and 400th epochs on the Cityscapes dataset and after the 100th and 150th epochs on the CamVid dataset.

### 4.3. Comparison with state-of-the-art algorithms

In order to evaluate the performance of our proposed method, we conducted a comparison with state-of-the-art approaches. In this section, we directly extracted their results from the original papers for comparison.

#### 4.3.1. Experiments on the Cityscapes dataset

On the Cityscapes dataset, we present the performance comparison between CCSONet and the state-of-the-art methods in Table 1. All these results are obtained using a single model on single-scale images. The symbol “–” indicates that the corresponding results were not provided by that method. In these comparisons, “R18” represents ResNet18, while “BiSeNet1 (Yu et al., 2018)” and “BiSeNet2 (Yu et al., 2021)” are two configurations with different network scales. According to the setup, DFANet (Li et al., 2019) has two versions, “Mode A” and “Mode B,” featuring distinct background channel settings.

**Table 1:** Comparison with real-time state methods on Cityscapes.

Model	GFLOPs	Params	FPS	mIoU
Enet (Paszke et al., 2016)	3.8	0.4	135.4	57.0
ICNet (Zhao et al., 2018)	28.3	26.5	30.3	69.5
BiSeNet1 (Yu et al., 2018)	14.8	5.8	105	68.5
BiSeNet2 (Yu et al., 2021)	55.3	49.0	45.7	74.7
DFANet A (Li et al., 2019)	3.4	7.8	100	71.3
DFANet B	2.1	4.8	120	67.1
ShelfNet (Zhuang et al., 2019)	–	14.8	59	74.8
SwiftNet (Wang et al., 2021)	114.0	12.9	34	75.1
FCHarDNet (Chao et al., 2019)	35.0	4.1	53	75.9
MGSeg (He et al., 2021)	54.3	13.3	84	76.4
CCSONet (R18, 768)	58.7	12.7	87	76.9
CCSONet (R18, 1024)	104.3	12.7	51	78.3

From Table 1, it can be observed that our proposed CCSONet excels in real-time segmentation networks. Regarding effectiveness and efficiency, CCSONet outperforms the current best real-time methods. Real-time methods like ENet (Paszke et al., 2016) and DFANet (Li et al., 2019) achieve high speeds in real-time scenarios, and their mIoU is only around 50–70%. While DFANet’s Mode A performs better, it is slower due to having more channels. ENet achieves a frame rate exceeding 130 FPS, but the accuracy is only 57 mIoU. SwiftNet (Wang et al., 2021), based on the encoder-decoder structure, has a larger model size and relatively higher computational costs. On the other hand, ShelfNet (Zhuang et al., 2019) improves performance through a lightweight decoder. The most competitive method is MGSeg (He et al., 2021), achieving 76.4 mIoU at a speed of 84 FPS, but it still lags behind CCSONet. Although most recent real-time methods use feature aggregation to enhance performance, CCSONet’s focus on small objects and context feature enhancement strategies yields even better results in performance improvement.

#### 4.3.2. Experiments on the CamVid dataset

To verify the generalization performance, we conducted experiments on the CamVid dataset, and the results are listed in Table 2. The input size for CCSONet was set to 720×960 . Overall, CamVid’s performance is slightly lower than that of Cityscapes, likely due to the higher image resolution in Cityscapes, making it more effective but less efficient. On the CamVid dataset, our proposed CCSONet stands out with a result of 73.1 mIoU and a speed of 138 FPS. Besides HRNetV2 (W48) (Wang et al., 2020) and DeepLabV3Plus + SDCNetAug (Zhu et al., 2019), CCSONet’s performance surpasses most methods. However, these two models are larger in scale and slower in speed. Although the efficiency of HRNetV2 (W48) was not reported in the original paper, its computational complexity exceeds 1200 GFlops, similar to DeepLabV3, making it less efficient. Compared to the real-time models BiSeNet and DFANet, CCSONet has advantages in both accuracy and speed. Compared to MGSeg, CCSONet improves the accuracy by 0.4 mIoU, indicating its potential for widespread application in various scenarios.

**Table 2:** Comparison with real-time state methods on Camvid.

Model	Time (ms)	FPS	mIoU
ENet	–	–	51.3
ICNet	36.0	28	67.1
BiSeNet1	–	–	65.6
BiSeNet2	–	–	68.7
DFANet A	8.3	120	64.7
DFANet B	6.3	160	59.3
FCHarDNet	6.7	149	67.7
MGSeg	7.9	127	72.7
HRNetV2 (Wang et al., 2020)	–	–	78.5
DeepLabV3Plus + SDCNetAug (Zhu et al., 2019)	–	–	81.7
CCSONet	7.2	138	73.1

### 4.4. Efficiency evaluation

The efficiency is a key factor in real-time semantic segmentation of the field. In previous studies, efficiency evaluation often depended on different hardware configurations, which could lead to unfair comparisons. We conducted re-experiments on the same hardware platform to ensure a fair comparison and employed official open-source code. In cases where official code was unavailable, we implemented nonofficial code and reported efficiency results in the paper, but these are for reference only. The accuracy is directly cited from the original paper, denoted by “#” in the table. We performed performance comparisons on the same hardware platform, and the results are summarized in Table 3. During inference, we set the batch size to 1. The reported efficiency (average FPS) is based on 1000 forward passes to maintain stability. Here, “X39,” “R18,” and “FCH70,” respectively, represent the use of Xception39, ResNet18, and FCHarDNet as backbone networks.

**Table 3:** Efficiency and accuracy comparison on Cityscapes.

Methods	Backbone	Input	GFlops	Params (M)	MAC (G)	FPS	mIoU
DFANet#	X39	1024 × 1024	3.4	7.8	0.98	33	71.3
BiSeNet#	R18	1024 × 2048	119	13.4	3.92	27	74.7
ShelfNet	R18	1024 × 2048	–	14.8	2.34	37	74.8
SwiftNet	R18	1024 × 2048	104	11.8	1.96	40	75.5
FCHarDNet	FCH70	1024 × 2048	35.4	4.1	3.60	53	75.9
MGSeg	R18	1024 × 2048	96.5	13.3	2.54	50	77.8
CCSONet	R18	768 × 1536	58.7	12.7	1.27	87	76.9
CCSONet	R18	1024 × 2048	104.3	12.7	2.46	51	78.3

Table 3 shows that DFANet exhibits poorer accuracy performance, mainly due to its rapid downsampling and late-stage attention strategies. Other real-time methods perform at around 75 mIoU and 40 FPS. DFANet and BiSeNet leverage private deep learning frameworks and optimize for deep convolutions on the hardware platform, achieving faster speeds. However, the nonofficial PyTorch code implementation only achieves around 30% of their originally reported speeds, highlighting the need for caution when using nonofficial code. Recent work such as MGSeg demonstrates superior performance. Our proposed CCSONet outperforms existing real-time methods, particularly showing slight improvement at the 768×1536 input resolution. When increasing the input size to 1024×2048 , CCSONet achieves significant improvements, with accuracy increasing by over 2 mIoU. This indicates CCSONet’s excellent adaptability to large-resolution inputs, potentially enhancing the performance of real-time semantic segmentation tasks.

**Figure 5:**
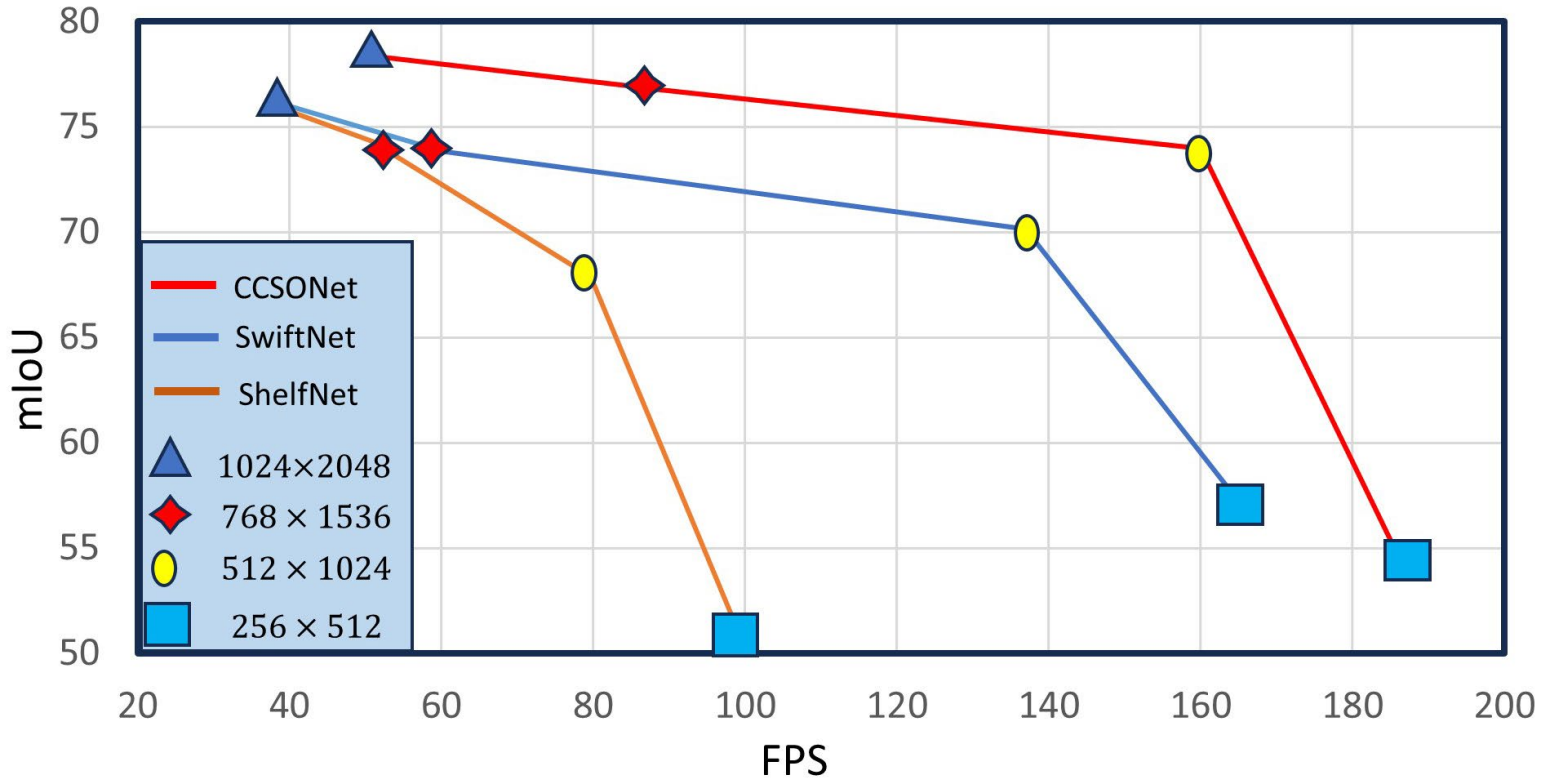
Effect of the speed given by different input resolutions on Cityscapes.

We further investigated the impact of segmentation model input size. Four input sizes were tested: 256×512,512×1024,768×1536 , and 1024×2048 . Comparison results between SwiftNet, ShelfNet, and CCSONet are illustrated in Figure 5. Larger sizes lead to slower speeds but better performance. However, when the input size exceeds 768 × 1536, the improvement becomes marginal. This is because other real-time models mostly rely on low-resolution feature maps, significantly losing small spatial details. Our designed network focuses more on small objects, making the performance drop less pronounced. Overall, CCSONet achieves better performance at resolutions from 512×1024 to 1024×2048 . Using smaller input sizes can improve speed. At an input resolution of 512×1024 , MGSeg achieves 73.9 mIoU and 160 FPS. Importantly, CCSONet is based on the widely used ResNet18 backbone network and implemented using PyTorch without any low-level optimizations.

We employ the concept of Memory Access Cost (MAC) to assess the memory utilization of the model. Specifically, MAC refers to the total memory accessed by the model’s weights and feature maps on the GPU. The significance of this metric is self-evident, as high memory access directly impacts the utilization of computational resources and the practical usability of the model. To gain a better understanding of MAC, let us take a look at its calculation formula:


(33) $$MAC=hw\left(c_{\mathrm{in}}+c_{\mathrm{out}}\right)+\frac{c_{\mathrm{in}}c_{\mathrm{out}}}{g}$$

Where h and w are the height and width, $c_{\mathrm{in}}$ and $c_{\mathrm{out}}$ are the input channels and output channels, and g represents the groups in the convolution layer. It is evident from Table 3 that memory access cost (MAC) is a key factor in accelerating the model. Despite SwiftNet and BiSeNet having similar FLOPs, but SwiftNet is faster compared to BiSeNet. This is because during the feature aggregation process, SwiftNet generates less feature maps than BiSeNet. At a similar MAC level, FCHarDNet’s FLOPs cost is significantly reduced compared to SwiftNet, but it only improves by 25. For lightweight networks, MAC is more important to the final inference speed than FLOPs. The proposed CCSONet requires only 1.27GMAC and 58.7GFLOPs, enabling it to achieve a speed of over 80 FPS.

### 4.5. Ablation experiments

#### 4.5.1. Contribution of individual components

Firstly, we conducted experiments to validate the contributions of LSCFEM and SOADM, as shown in Table 4. Without LSCFEM and SOADM, the performance of ResNet18 is notably poor, achieving only around 67 mIoU. Due to the smaller scale of ResNet18, segmentation utilizes only 1/32 of the features, resulting in a significant loss of details. When employing LSCFEM, performance improves, indicating the effectiveness of LSCFEM. The combination of LSCFEM and SOADM further enhances performance. The contribution of SOADM is particularly significant, facilitating the segmentation of small objects while maintaining similar efficiency. An improvement of 1.4 mIoU is achieved with high-resolution inputs, owing to the larger resolution providing more accurate details in multiscale features, enriching the final results.

**Table 4:** Effect of individual component of CCSONet.

ResNet18			768 × 1536		1024 × 2048	
			FPS	mIoU	FPS	mIoU
√			110	67.1	68	69.0
√	√		103	75.1	62	76.1
√	√	√	87	76.9	51	78.3

#### 4.5.2. Effectiveness of LSCFEM

##### 4.5.2.1. Region relevance visualization

**Figure 6:**
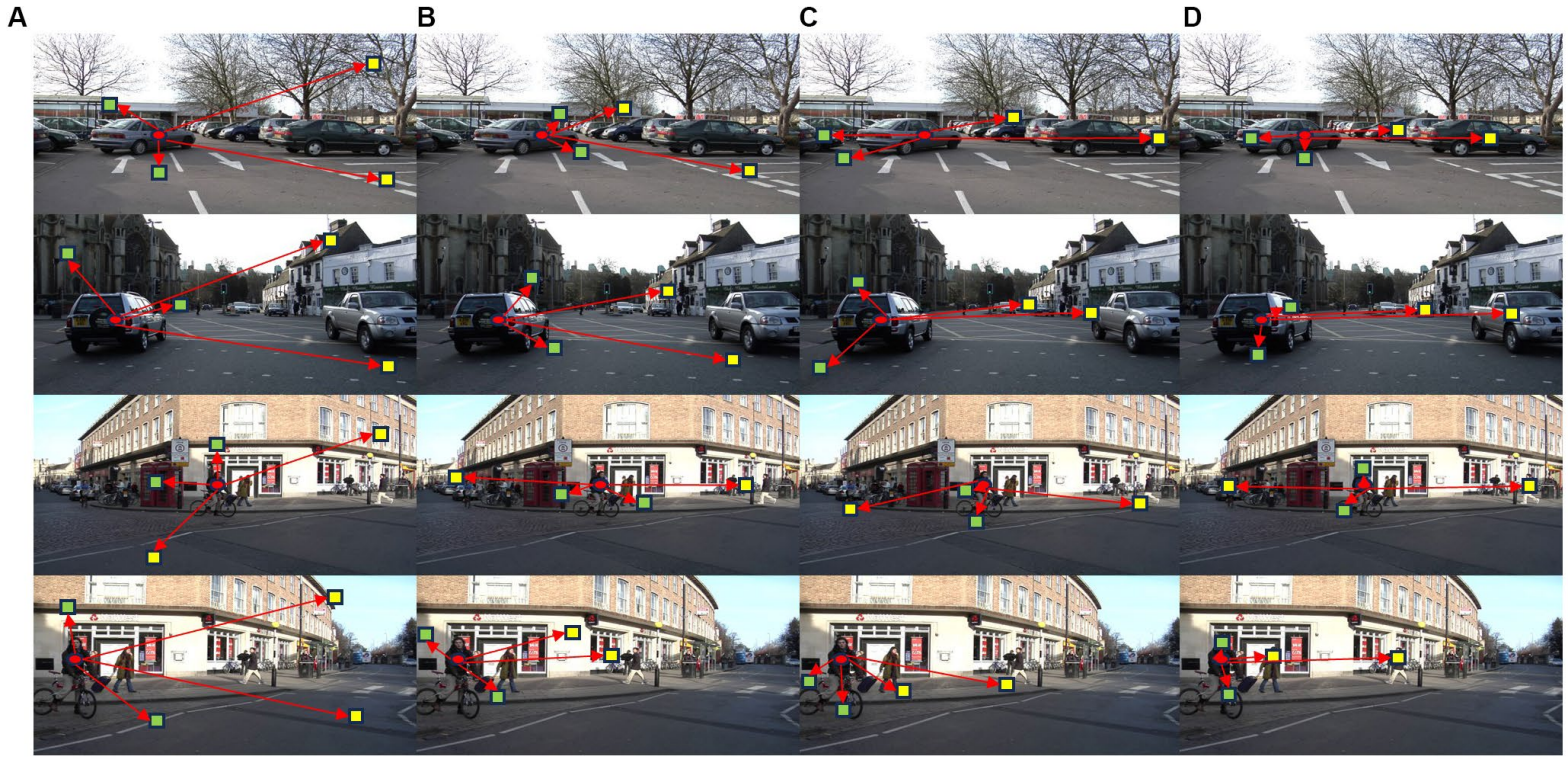
The evolution of related regions of the same pixel during the learning process.

Figure 6 displays the evolution of four related regions of the same pixel during the learning process. We selected two short-range related regions and two long-range related regions. The first column (Figure 6A) illustrates the state of these regions during random initialization. From the second column to the last column (Figures 6B–D), we used three networks trained with 10 K, 30 K, and 50 K mini-batches, respectively, to compute the related regions for the selected pixel.

The short-range and long-range related regions cover local and longer-range content, demonstrating that the related regions capture context from different scales. In the top and bottom rows of Figure 6, we compared two images with similar scenes, demonstrating the similarity in the distribution of related regions for the same category. For instance, the closely related vehicle regions include the vehicle and the road near its edge, while the distantly related regions encompass other vehicles.

In the early stages of network training, the positions of the short-range and long-range related regions are distributed across the entire image, showing limited correlation. As the network training stabilizes, the short-range and long-range related regions in similar scenes gradually align with objects that share semantic and spatial relationships, forming a coherent context. These observations reveal the evolving process of the model’s understanding of images and feature learning during the training process.

##### 4.5.2.2. Number of related regions

**Figure 7:**
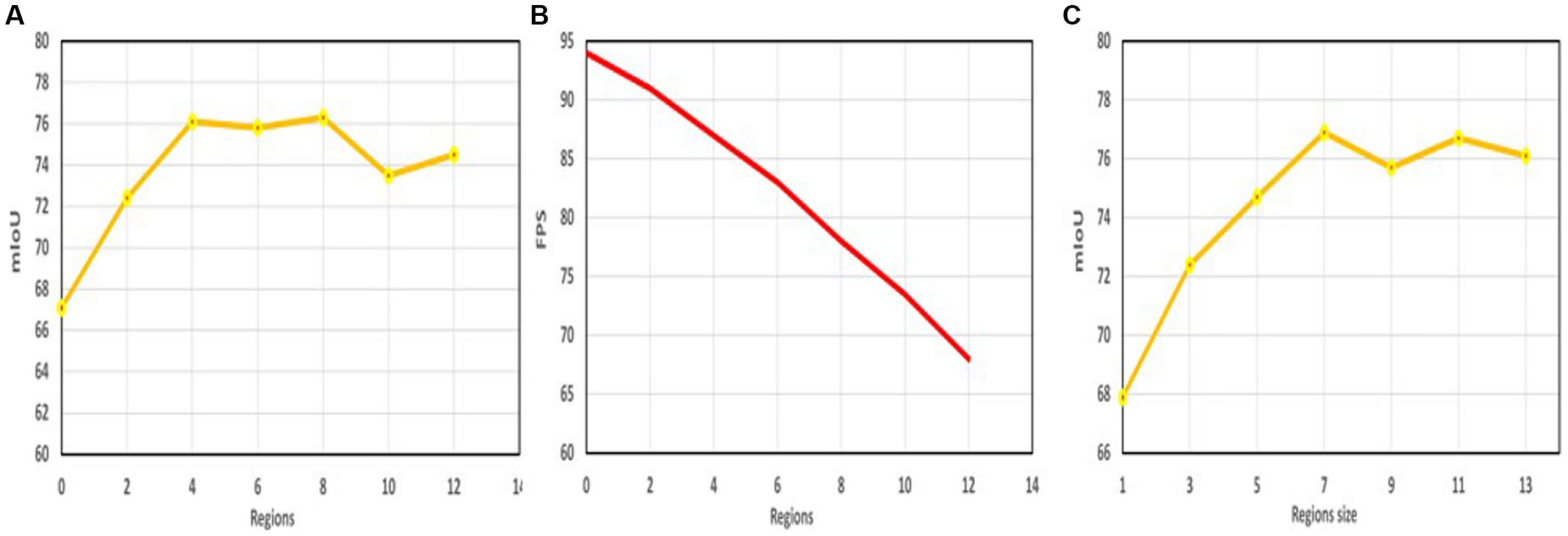
**(A)** The relationship between the number of relevant regions and the accuracy, **(B)** The relationship between the number of relevant regions and the speed, **(C)** The relationship between the size of the relevant region and the accuracy.

In Figure 7A, we evaluate segmentation accuracy on the Cityscapes validation set using different numbers of related regions ( $T\in \left\{1,2,4,6,8,10,12\right\}$ ), with other region hyperparameters set to default values and an input resolution of 768×1536 . T=1 indicates no configurable regions are used.

When T=1 , without the context enhancement provided by LSCFEM, the network only achieves 67.1 mIoU. As the number of related regions increases, segmentation performance significantly improves. For example, at T=4 , the network achieves an accuracy of 76.9 mIoU, highlighting the effectiveness of LSCFEM. However, we observe that with even more regions, such as T=8,10 , the network’s performance does not improve further. This is due to increased complexity from additional regions, making it harder to train the CCSONet network with limited data. It’s important that more regions also require more computational costs, such as GPU memory, model parameter count, and FLOPS. Figure 7B shows the relationship between network running speed and related region settings; as the number of related regions increases, network speed decreases. In this study, T=4 strikes the best balance between performance and efficiency for CCSONet.

##### 4.5.2.3. Size of related regions

In Figure 7C, we conducted segmentation experiments using different region sizes (including 1×1,3×3,5×5,7×7,9×9,11×11 , and 13×13 ), with an input resolution of 768×1536 . The results suggest that larger regions with richer contextual information may lead to higher mIoU. However, if the region size becomes too large, the configurable regions in LSCFEM can overlap and capture irrelevant image content, resulting in negligible improvement in segmentation accuracy but increased computational costs. In our experiments, the size of configurable regions did not affect GPU memory requirements, as related region pooling and upsampling operations generate feature maps of the same size, making little difference in GPU memory usage.

#### 4.5.3. Effectiveness of SOADM

##### 4.5.3.1. Enhancing small object detection

To demonstrate the effectiveness of the proposed SOADM, we evaluate the module using four widely used segmentation networks and the CCSONet itself. These four networks can be categorized as follows: (1) LinkNet, a model based on a fully convolutional encoder-decoder architecture; (2) Unet, which utilizes skip connections to fuse low-level and high-level features; (3) PSPNet, a model based on pyramid pooling and dilated convolutions; and (4) PAN, which incorporates attention mechanisms. This experiment aims to validate the SOADM module’s assistance in small object segmentation and show its generalization capability in other types of semantic segmentation networks.

Table 5 presents the evaluation results on the CamVid dataset. We define symbols, pedestrians, poles, and bicycles as small objects and the rest as large objects. Applying the SOADM module to the baseline segmentation networks significantly improves the accuracy scores for small objects compared to models without SOADM. Combining the SOADM module with Unet, PAN, LinkNet, PSPNet, and CCSONet segmentation networks improves segmentation accuracy for the small object category. Table 5 also shows that applying SOADM to Unet, PAN, LinkNet, PSPNet, and CCSONet slightly improves the large object segmentation, but the improvement could be more pronounced.

**Table 5:** The comparison results of small object classes and large object classes on Camvid.

Models	Small object				Large object							mIoU
	Signsymbol	Pedestrian	Pole	Bicyclist	Building	Tree	Sky	Car	Road	Pavement	Fence	
Unet	52.1	57.0	38.5	53.8	80.9	74.3	91.5	83.4	93.0	79.4	44.2	68.0
Unet+SOADM	54.4	59.4	41.4	63.9	81.9	76.3	91.8	86.2	92.7	79.5	50.1	70.7
PAN (Li et al., 2018)	51.4	49.5	38.1	57.8	79.7	74	89.9	79.8	93	80.4	42	66.9
PAN+SOADM	54.9	59.7	39.6	65.4	82.1	76.1	90.4	85.8	94.5	82.4	49.1	70.9
LinkNet (Chaurasia and Culurciello, 2017)	51.2	52.7	38.3	64.3	80.2	75.9	92.1	85.2	93.6	81.3	40.8	68.7
LinkNet+SOADM	54.1	61.8	42.4	65.4	81.7	76.1	92.4	85.9	93.9	81.1	50.1	71.4
PSPNet (Zhao et al., 2017)	50.0	54.0	36.6	61.1	74.5	69.8	90.7	80.0	89.6	79.2	46.1	66.5
PSPNet+SOADM	55.3	57.1	38.1	61.9	82.9	76.7	90.9	84.5	93.1	80.1	50.9	70.1
CCSONet	55.1	56.5	37.6	61.5	82.1	76.8	89.7	84.1	92.9	79.8	50.7	69.7
CCSONet+SOADM	56.5	64.4	44.2	66.4	84.5	78.7	92.7	87.5	95.2	82.6	51.3	73.1

##### 4.5.3.2. Visualization and understanding of SOADM

**Figure 8:**
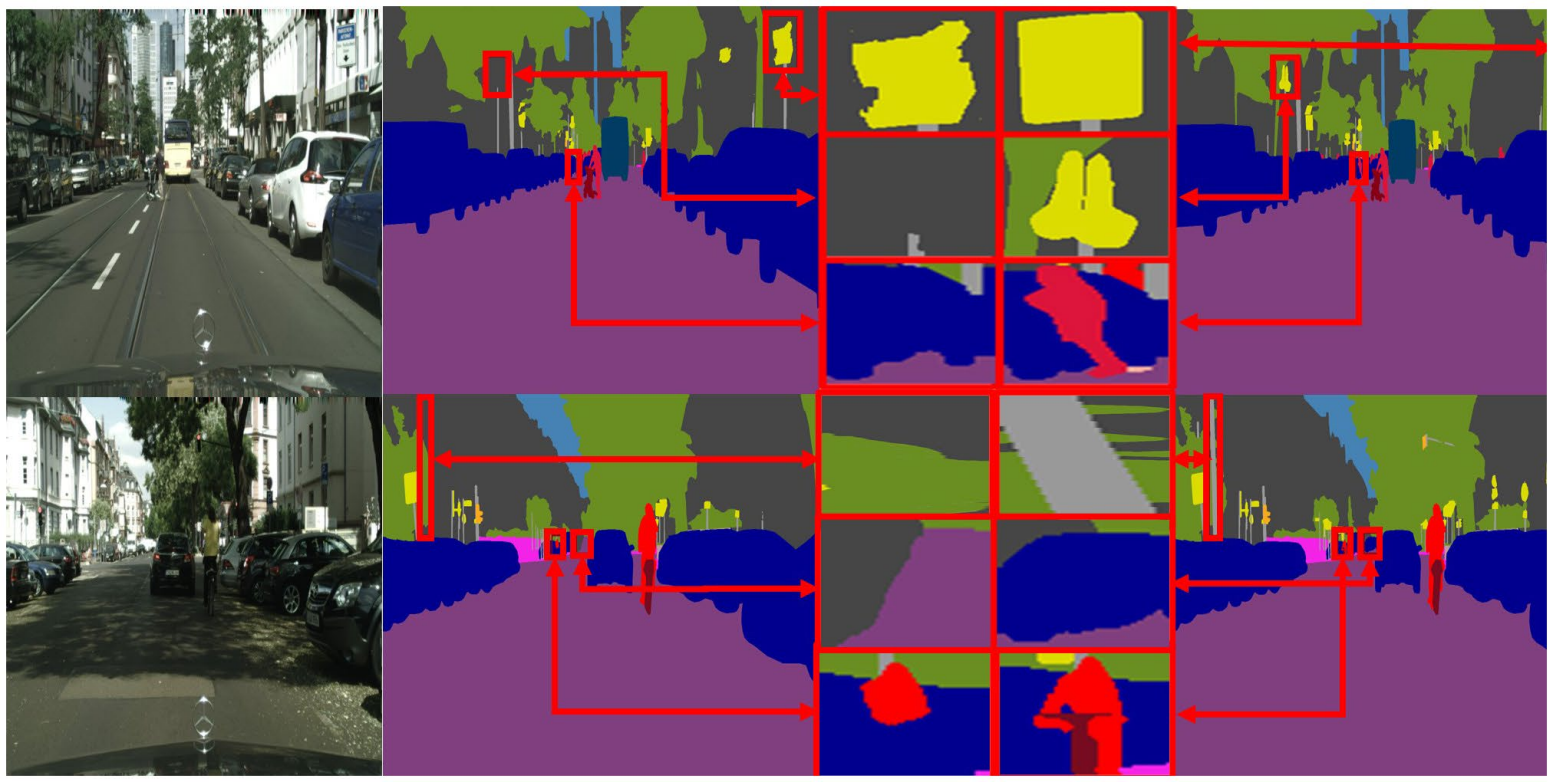
Visualization results of SOADM. From left to right are: original image, label, CCSONet without SOADM, detail comparison figure and CCSONet with SOADM.

To visually demonstrate the effectiveness of the proposed SOADM, Figure 8 shows representative segmentation results using CCSONet with and without SOADM on the Cityscapes test set. These figures illustrate that SOADM-based methods can achieve higher accuracy for small object classes such as cars, utility poles, and symbols. The small targets are too small in the segmentation result image, making it impossible to compare clearly. We enlarge the small targets in the segmentation result and connect the corresponding enlarged images with red lines. For a clear comparison, we put the magnified images of small targets at the same location in the same row. The comparison results in columns 3 and 4 of Figure 8 clearly show that the SOADM module network can segment pedestrians, vehicles, and symbols in the distance. These cases show that our SOADM can improve the network’s segmentation accuracy for small targets. The main mechanism of this module is to learn the correlation between the target object and related objects and use the characteristics of related objects to complete the missing characteristics of small target objects. Figure 8 further illustrates that SOADM can better complete the missing information of small objects and generate more accurate segmentation results.

##### 4.5.3.3. Performance based on pixel size groups

The evaluated models above classified object sizes based on object categories into small/large groups. However, objects belonging to large object categories (such as cars) may appear as small objects at a distance and vice versa. In this section, we redefined small/large objects based on pixel sizes and conducted evaluations accordingly. We performed pixel size statistics for each object category in the Camvid dataset, defining objects with sizes smaller than 32×32 pixels as small targets, those larger than 96×96 pixels as large targets, and the rest as medium targets. According to these results, the “small group” includes symbols, utility poles, pedestrians, cyclists, and fences; the “medium group” comprises sidewalks, trees, and cars; the “large group” consists of sky, buildings, and roads.

Table 6 presents the quantitative results of the Camvid dataset. Models based on SOADM outperform the baseline method in segmenting small objects. For instance, when combined with SOADM, Unet, PAN, LinkNet, PSPNet, and CCSONet, achieve improvements of 4.2, 4.2, 3.8, and 4.5% in small object semantic segmentation. Similar results are shown in Tables 5, 6. Regardless of the definition criteria based on small/large objects or pixel sizes, SOADM significantly enhances the performance of the baseline model on small objects. Small or large objects grouped by category or pixel size exhibit similar object distributions. The definition of small objects based on object pixels is consistent with the definition based on object categories, wherein most symbols, utility poles, pedestrians, and cyclists are objects with relatively small pixel dimensions.

**Table 6:** The comparison results of small / non-small object grouped by pixel size on Camvid.

Model	Object size		
	Small	Medium	Large
Unet	15.7	35.7	84.6
Unet+SOADM	19.9	37.8	86.4
PAN	13.9	34.1	84.2
PAN+SOADM	18.1	38.0	85.9
LinkNet	15.1	34.9	84.8
LinkNet+SOADM	18.9	37.7	85.6
PSPNet	14.7	30.1	81.0
PSPNet+SOADM	19.2	33.1	81.7
CCSONet	18.6	33.2	81.3
CCSONet+SOADN	22.7	36.3	84.8

### 4.6. Case study

**Figure 9:**
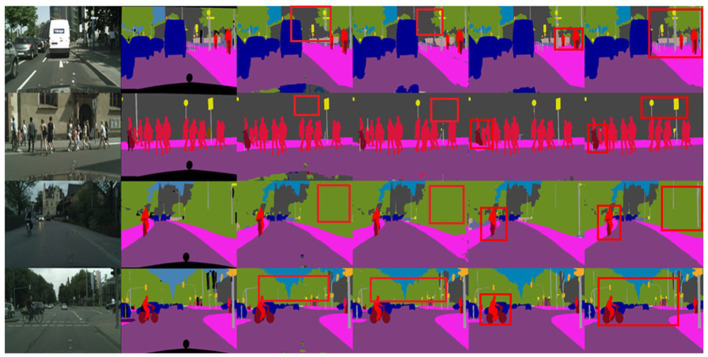
Segmentation visualization results plot. From left to right: BiSeNet, SwiftNet, MGSeg, and CCSONet.

Figure 9 presents the results of four segmentation methods: BiSeNet, SwiftNet, MGSeg, and CCSONet. The first column of these images displays the original images, while the second column shows the ground truth label information. Overall, these methods can relatively accurately identify the outlines of the street scene images. However, there are still some details that are challenging to classify accurately. For example, utility poles are mistakenly identified as walls or trees in the segmentation by BiSeNet and SwiftNet.

In contrast, our proposed CCSONet can recognize boundary details and control segmentation granularity, successfully classifying these intricate details. Additionally, compared to the MGSeg method, CCSONet performs more accurately in boundary segmentation of objects such as bicycles and pedestrians. This result further underscores the superiority of CCSONet in image segmentation tasks.

## 5. Conclusion

This paper proposes a lightweight semantic segmentation network, the Configurable Context and Small Object Attention Network (CCSONet), which explores the potential correlations between small and large objects while studying the effects of long and short-range contextual information on feature enhancement. We design the long-short distance configurable context feature enhancement module (LSCFEM) to address feature distortion during the encoding process and introduce the small object attention decoding module (SOADM) to enhance the segmentation accuracy of small objects. We extensively analyze and quantitatively experiment on the standard datasets Cityscapes and CamVid, providing strong validation for the effectiveness of our proposed CCSONet. Our approach significantly outperforms existing state-of-the-art methods, introducing new directions and possibilities for advancing the field of image segmentation. In future research, we will explore weakly supervised solutions, which will further improve segmentation accuracy, particularly in boundary segmentation. Additionally, we will focus on detail encoding methods for low-resolution feature maps, enhancing the robustness and applicability of our method across various practical scenarios.

## Data availability statement

Publicly available datasets were analyzed in this study. This data can be found here: All experiments are reproducible and the datasets we use are: Cityscapes and CamVid. The official URLs are https://www.cityscapes-dataset.com/ and http://mi.eng.cam.ac.uk/research/projects/VideoRec/CamVid/.

## Author contributions

CZ: Conceptualization, Data curation, Resources, Software, Writing – original draft. FX: Software, Writing – review & editing. CW: Visualization, Supervision, Writing – review & editing. JL: Software, Writing – review & editing, Formal analysis, Visualization.
